# Treatment preferences of patients with muscle invasive bladder cancer: A discrete choice experiment

**DOI:** 10.1002/bco2.443

**Published:** 2024-10-11

**Authors:** Liam Mannion, Verity Watson, Vinod Mullassery, Rajesh Nair, Thomas Charlton, Margaret Northover, Deborah Enting, Mieke Van Hemelrijck, Muhammad Shamim Khan, Ramesh Thurairaja, Suzanne Amery, Kathryn Chatterton, Kate Smith, Simon Hughes

**Affiliations:** ^1^ City University of London London UK; ^2^ King's College London London UK; ^3^ Guy's and St Thomas' NHS Foundation Trust London UK; ^4^ University of Aberdeen Aberdeen UK; ^5^ The Royal Marsden NHS Foundation Trust London UK

**Keywords:** cancer treatment preferences, discrete choice experiment, muscle invasive bladder cancer, patient choice

## Abstract

**Background:**

When faced with treatment options, patients are asked to participate in decision‐making. We sought to determine which treatment aspects matter most for individuals treated for muscle invasive bladder cancer (MIBC), with an aim to improve understanding of patient preferences and what trade‐offs patients are willing to accept. Our study consisted of a discrete choice experiment (DCE): a type of questionnaire used to elicit preferences in the absence of real‐world choice.

**Methods:**

The DCE had five attributives, each with three levels. Participants were asked to complete a questionnaire in which they were asked to choose between two hypothetical MIBC treatments. The data were analysed using a conditional logit model, and preferences for, and trade‐offs between, attributes were estimated.

**Results:**

We recruited patients with MIBC who had either already completed, were undergoing or had yet to commence radical treatment for MIBC (*n* = 60). Participants indicated a strong preference for treatments that increased their life expectancy (*p* = <0.001), had a lower risk of long‐term complications (*p* = <0.001) and less changes to their body image (*p* = <0.001). Changes to sexual wellbeing (*p* = 0.09) or an increase in acute side effects (*p* = 0.99) did not influence preferences. Patients were willing to accept treatments with higher risk of long‐term complications to improve their life expectancy or body image.

**Conclusion:**

When deciding on the type of treatment, increased life expectancy is the most important consideration for people with MIBC. The risk of long‐term complications and changes to overall body image as a result of treatment are also important. Our study also highlighted that patients are willing to accept a higher risk of long‐term complications to improve other treatment outcomes. Understanding patient preferences is important for shared decision‐making, which has an impact on quality of care for people living with MIBC.

## 1 INTRODUCTION

Radical treatment for muscle invasive bladder cancer (MIBC) involves making decisions regarding the use of systemic chemotherapy, removal of the bladder with a urinary diversion or bladder preservation with (chemo)radiotherapy. Decisions are made based on tumour factors (histological subtype, tumour grade, concomitant non‐muscle invasive bladder cancer [NMIBC]) and patient factors (preferences, comorbidities, previous treatments, fitness, bladder function). This involves a complex decision‐making process, and there is limited information available regarding patient preferences and acceptable trade‐offs to help guide support for the process. For example, it has been suggested that older patients may prioritise quality of life over overall or progression‐free survival.^1^ Informed patients may also experience less treatment decision regret, for example, patients who were more informed on bladder reconstruction options experienced less decision regret post radical cystectomy (RC).^2^

Quantifying patient healthcare preferences is key to determining patient centric healthcare policies, designing clinical trials with outcomes that are important to patients and developing educational content appropriate for informed decision‐making. In a resource constrained healthcare system, better understanding of patient preferences can guide the prioritisation of resources towards outcomes that patients value. Considering this, the NHS and National Institute for Health and Care Excellence (NICE) plan to integrate patient‐centred evaluation into their future technology assessments, to compliment the existing clinical and cost effectiveness assessments.^3^, ^4^ Clinical trials are often designed with end‐points determined by clinicians/scientists and/or industry. Whilst this has played a key role in evolving patient care, it has led to a focus on measurable outcomes (often surrogates), which have limited meaning to patients. A greater focus on what is important to patients will guide the development of more relevant studies. A clear understanding of treatment outcomes is also important for patients to guide their decision‐making process.^5^, ^6^, ^7^ Patients need to balance the expected toxicity of treatment against the predicted benefits in outcomes. Shared decision‐making is key to this assessment, with the clinical teams supporting patients through the process. A good, shared decision is one where the patients are well‐informed about the options, understand what matters most to them and make a decision that is consistent with their values. Information needs to be designed for patients that facilitates this process and focusses on what they find important.

The discrete choice experiment (DCE) is a method used in health services research to explore how people prioritise and trade‐off between different treatment outcomes in a constrained manner.^8^ DCEs have been increasingly used to elicit patient preferences in oncology, most commonly in breast, prostate and oesophageal cancer.^9^, ^10^, ^11^, ^12^, ^13^ Given that each tumour site brings unique complexities regarding treatment options, patient demographics and preferences, primary research is needed for each cancer.

The primary objective of this study was to gain a greater understanding of the treatment preferences and the trade‐offs for patients when making radical treatment decisions in MIBC.

## 2 METHODS

The study was conducted using validated and established protocols for undertaking DCEs.^11^, ^14^ The study was approved by the NHS Health Research Authority (Integrated Research Application System number: 282974) and registered at https://clinicaltrials.gov/study/NCT05236218. Additional ethical approval was granted by Kings College London (ethical review reference number MRSP‐20/21‐21687) to conduct focus groups with healthcare professionals specialising in the management of bladder cancer, and patients who have previously received a diagnosis of MIBC.

The attributes and levels included in this DCE were informed by a best practice approach that combined a literature review and formative qualitative research. A literature review of peer reviewed publications identified the most important considerations for patients when making treatment decisions for MIBC. Relevant articles were identified through PubMed and Google scholar using search terms *Muscle Invasive Bladder Cancer*, *MIBC* and *patient preferences* or *treatment outcomes* or *treatment preferences*. A summary of the articles included in the review is within Appendix A. The considerations were collated into a list of attributes for discussion within the qualitative focus groups.

The focus groups were organised to determine the most important treatment attributes that should be included in the DCE. There were two clinician‐based focus groups (Group 1: *2× clinical oncologist*, *1× medical oncologist*, *1× urology clinical nurse specialist*; Group 2: *1× urologist*, *1× urology clinical nurse specialist*) and one patient‐based focus group (five patients). The initial set of attributes was derived from the literature review, but it was also possible to add new attributes if these had not been identified in the review. The clinical focus groups were also tasked with providing a range of levels for the clinically relevant outcomes for each attribute. The final DCE questionnaire included five treatment attributes each with three levels (Table 1).

**TABLE 1:** Attributes and levels used in the discrete choice experiment (DCE).

Attribute	Description	Levels
Body image	Changes in body image/appearance caused by the treatment. Possible changes include a stoma, an external bag that collects urine or scarring	• Unchanged (no visible change) • Slightly changed • Significantly changed
Life expectancy	Life expectancy following treatment with regard to mean survival in MIBC	• Strong increase • Moderate increase • Not increased
Side effects from treatment	Treatments for bladder cancer often cause side effects. Acute side effects are problems that occur when the treatment affects healthy tissues or organs. Occur during the treatment, and typically go away a few weeks after treatment is finished. They may include …., and side effects specific to the area being treated. However, in some case, acute side effects may be more serious	• Eight of 20 patients will have a complication during their treatment that will require hospitalisation • Ten of 20 patients will have a complication during their treatment that will require hospitalisation • Twelve of 20 patients will have a complication during their treatment that will require hospitalisation
Living as bladder cancer survivor (long‐term complications)	Living as bladder cancer survivor: Patients who are successfully treated for bladder cancer are at risk of developing complications months or years after their treatment. Most side effects gradually go away in the weeks or months after treatment. But some side effects can continue. Or you might notice some that begin months or years later.	• Three out of 20 patients who underwent treatment had at least one long‐term complication from their treatment. • Five out of 20 patients who underwent treatment had at least one long‐term complication from their treatment • Eight out of 20 patients who underwent treatment had at least one long‐term complication from their treatment
Sexual wellbeing	Many patients with bladder cancer may experience changes to their sexual wellbeing—they may include changes in the way you feel about your body and how you feel about having sex. This may be caused by the cancer itself or by treatments for the bladder cancer.	• No change in sexual wellbeing • Reduced sexual wellbeing in comparison to before the treatment • A complete loss of sexual wellbeing

Abbreviation: MIBC, muscle invasive bladder cancer.

**FIGURE 1:**
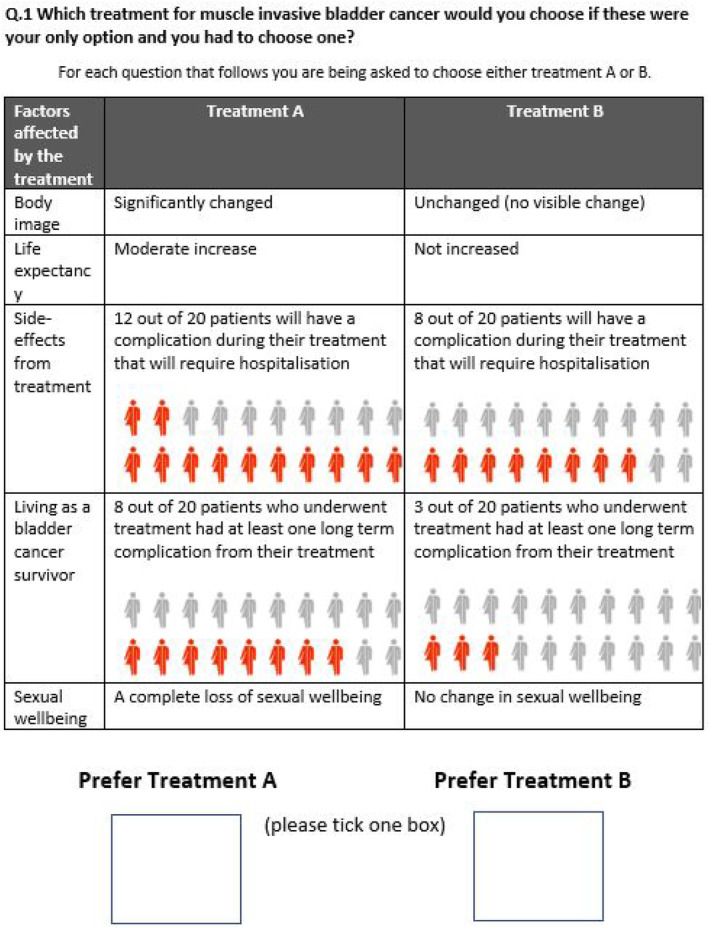
Example of choice set used in the final discrete choice experiment.

The attributes and levels combine into 243 different treatment alternatives and 29 403 possible pairs of alternatives. We reduced this to a manageable number of 15 choice tasks using a D‐efficient experimental design with small directional priors for a main effects only model using Ngene software.^15^ Each choice task consisted of 15 pairs of hypothetical clinical scenarios (choice sets), each with different clinical outcome levels—for patients to select their preferred option from each pair. One set of questions was generated, and all participants considered the same hypothetical treatments. See Figure 1 for an example of one of the questions.

There is a lack of published data to inform sample size calculations for healthcare‐related DCEs, but even a small sample size can provide meaningful data.^16^ Considering the prevalence of MIBC in our bladder cancer clinic, we opted to recruit 60 participants in total over 12 months. Recruitment was planned in two cohorts: Cohort 1 (the pilot study) recruited 10 patients. In addition to completing the questionnaire, these patients were also asked to participate in a brief unstructured interview to discuss the clarity of the questionnaire and their understanding of the clinical scenarios in each choice set. This led to the incorporation of an additional explanation for each choice set within the final DCE questionnaire. Cohort 2 consisted of 50 patients. All patients were recruited from a weekly specialist multidisciplinary bladder cancer clinic at a tertiary referral centre. Patients were eligible for recruitment if they had received a diagnosis of MIBC suitable for radical treatment—for example, they could be newly diagnosed, undergoing radical treatment, undergoing routine follow‐up or have been diagnosed with recurrent disease following initial radical therapy. The study was therefore undertaken in a relatively fit population (WHO performance status 0–2); all patients over 65 with comorbidities were reviewed by a specialist geriatric oncology team for optimisation (this is standard of care at our institution irrespective of the management approach selected), and we did not include patients with functional dependence, significant comorbidities or frailty.

A conditional logit model was estimated in STATA™ (version 17.0). The model estimated the relative importance of the attributes and levels to participants' choice of MIBC treatment. The model is based on random utility theory and assumes that participants (n) choose the treatment (j) that provides the highest utility in each choice task (t). The utility of a treatment (V_njt_) is a linear and additive function of the treatment attributes and levels. In order to assess the overall preferences of our participants, a utility ranking based on each relative attribute importance (RAI) was calculated.

We calculated the values in two different ways:

1. Increase from the lowest level (no change) to the moderate level (e.g., for body image, this would be significantly changed to slightly changed)
2. Increase from the lowest level (no change) to the highest level (e.g., for body image, this would be no change to significantly changed).

We also calculated trade‐offs on the attributes with continuous variables (levels described with numerical values). In this DCE, there were two such attributes: acute side effects and long‐term complications.

## 3 RESULTS

The most commonly appearing factors in the literature were survival (overall and cancer specific), bladder preservation versus urinary diversion and complications from treatment (acute and chronic) including the impact on sexual function/wellbeing post treatment. These were collated into a list of attributes for discussion within dedicated focus groups.

The focus groups refined these considerations into five main treatment attributes, with corresponding levels for analysis (see Table 1):

- Body image
- Life expectancy
- Significant side effects (acute) from treatment requiring hospitalisation
- Bladder cancer survivorship (chronic long‐term side effects from treatment)
- Sexual wellbeing

Patient recruitment took place within a single dedicated weekly multidisciplinary bladder cancer clinic at a tertiary referral centre. Between 17 June 2022 and 16 June 2023, 649 patients were screened for eligibility, of which 218 were eligible, and 79 were approached. Nineteen patients declined participation. Study recruitment was in two cohorts: phase 1: 17 June 2022–25 November 2022: a pilot study (*n* = 10); and phase 2: 2 December 2022–16 June 2023: recruitment of the remaining 50 patients. Patient characteristics are shown in Table 2: Median age was 69 years old; 80% were male; most were stage T2N0 (66.7%); 10% were pre‐treatment; 63% on treatment (self‐reported by participants); and 27% were on follow‐up post radical therapy or receiving active treatment for recurrent disease.

**TABLE 2:** Participant characteristics who completed the discrete choice experiment (DCE) (*n* = 60).

Characteristics	No. of patients (%)	
	Mean	SD
Age (years)	69.5	10.5
Age group		
˂40	1 (1.5)	
40–49	0 (0)	
50–59	8 (13.5)	
60–69	15 (25)	
70–79	24 (40)	
80–89	12 (20)	
90+	0 (0)	
Gender		
Male	48 (80)	
Female	12 (20)	
Clinical stage		
T2N0M0	40 (66.7)	
T3N0M0	11 (18.3)	
T3N1M0	4 (7)	
T4b	1 (1.5)	
T4N1M0	3 (5)	
Unknown (at least T2)	1 (1.5)	
Treatment status		
Had not started treatment but was due to	6 (10)	
Currently undergoing treatment	38 (63)	
Had completed treatment (in follow‐up)	16 (27)	

**FIGURE 2:**
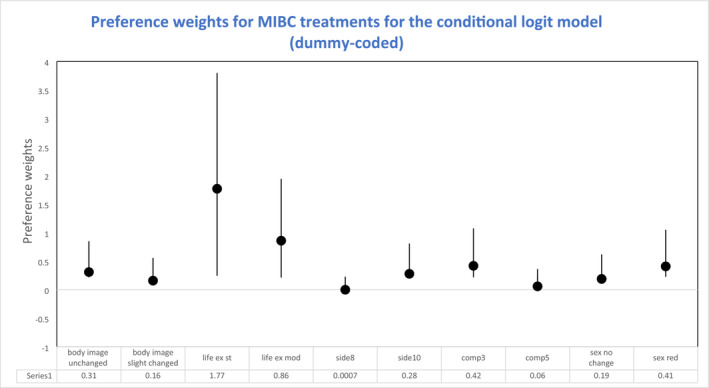
Preference weights for muscle invasive bladder cancer (MIBC) treatments using the conditional logit model (dummy‐coded) (*n* = 60).

Participants indicated a strong preference for treatments that increased their life expectancy (*p* = <0.001), resulted in fewer changes to their body image (*p* = <0.001) and had lower risk of long‐term complications (*p* = <0.001). The likelihood of acute side effects (*p* = 0.99) or changes to participants' sexual wellbeing (*p* = 0.09) did not influence treatment preferences (Figure 2).

Table 3 presents the RAI: Life expectancy was most important (0.91), followed by incidence of long‐term complications (0.37) and impact on body image (0.15). Impact on sexual wellbeing (−0.21) and acute side effects (−0.29) did not influence treatment decisions. Participants were willing to accept treatments with a higher risk of long‐term complications if it improved their life expectancy, body image or sexual wellbeing. We were able to calculate the degree to which participants were willing to trade‐off or their willingness to accept an increase in long‐term complications to improve their body image, life expectancy and sexual wellbeing (Table 4).

**TABLE 3:** Utility score and preference ranking of each attribute (*n* = 60).

Attribute	Coefficient (highest)	Coefficient (medium)	Utility score	Relative importance ranking
Life expectancy	1.78	0.87	0.91	1
Long‐term complications	0.43	0.06	0.37	2
Body image	0.32	0.17	0.15	3
Sexual wellbeing	0.20	0.41	−0.21	Did not influence treatment preferences
Acute side effects	0.00	0.29	−0.29	Did not influence treatment preferences

**TABLE 4:** Treatment preferences for patients with muscle invasive bladder cancer, conditional logit model with trade‐offs for long‐term complications (*n* = 60).

Attribute	Coefficient	Standard error	*p* value (significant^a^)	Long‐term complications (trade‐off willingness)	% accepted to improve from REF to best or middle level
Changes in body image/appearance caused by the treatment					
Unchanged (no visible change)	0.281	0.109	0.00^a^	−3.2	16%
Slight change	0.22	0.116	0.05^a^	−2.6	13%
Significant change	REF.				
Life expectancy following treatment with regard to mean survival in MIBC					
Strong increase in life expectancy (60% of people survive for 5 years after treatment)	1.777	0.129	0^a^	−20.8	100%
Moderate increase in life expectancy (55% of people survive for 5 years after treatment)	0.865	0.111	0^a^	−10.1	50%
Not increased (50% of people survive for 5 years after treatment)	REF.				
Side effects from treatment (converted to continuous variable)					
8/20, 10/20, or 12/20 *will* have a side effect (complication) that requires hospitalising but will still be able to continue with their treatment	0.000	0.028	0.98	‐	‐
Living as a bladder cancer survivor—long‐term complications (converted to continuous variable)					
3/20, 5/20, or 8/20 who underwent treatment *had at least one* long‐term side effect/complication from their treatment	−0.085	0.022	0.00^a^	‐	‐
Sexual wellbeing					
No change	0.198	0.117	0.09	−2.5	12.5%^a^
Reduced sexual wellbeing	0.411	0.116	0^a^	−4.7	23.5%^a^
Complete loss of sexual wellbeing	REF.			‐	

*Note*: The values in the column *Long‐term complications are* the trade‐off values, % differences/0.085 when selecting for individual attributes. For example, −10.17 represents a risk level of 10/20 who underwent treatment that had at least one long‐term side effect/complication from their treatment. Conditional (fixed‐effects) logistic regression/Log likelihood = −500.44231; number of obs = 1800; LR chi2 = 279.22; Prob > chi2 = 0.0000; pseudo R2 = 0.2181.

Abbreviations: MIBC, muscle invasive bladder cancer; REF, reference.

^a^ Although sexual wellbeing was significant (when assessed as a single observation) for reduced sexual wellbeing, we need to ignore this, as it does not make logical sense (e.g., they prefer the middle level to the highest level).

Patients were willing to accept the following:

- 100% risk of a chronic long‐term condition to achieve a 10% increase in life expectancy.
- 50% risk of a chronic long‐term condition to achieve a 5% increase in life expectancy.
- 16% risk of a chronic long‐term condition to avoid a significant change in body image.
- 13% risk of a chronic long‐term condition to avoid a slight change in body image.

The full results of the DCE are presented in Table 4.

## 4 DISCUSSION

DCEs are a useful tool for predicting real‐world behaviours and preferences regarding healthcare decisions.^17^, ^18^ Current practice is driven by disease outcomes and health economics. Whilst these are very important parameters, patient preference and outcomes need to be taken into consideration in the decision‐making process. DCEs have been undertaken for NMIBC. For example, one study found that patients who are unresponsive to Bacillus Calmette‐Guérin (BCG) were willing to make substantial benefit–risk trade‐offs to delay RC, such as accepting a 43.8% risk of progression and a 66.1% increase in the risk of serious side effects, with the latter having the least influence on treatment preferences.^19^ This is the first DCE conducted to assess patients' priorities and trade‐offs when considering radical treatment options for MIBC.

We used a utility maximisation technique to determine factors that are important to patients when making shared management decisions. Assessing the relative importance between the medium and highest level of our attributes, the results demonstrate that treatments offering a greater increase in survival yield 5.7 times (1.77/0.31) as much utility as treatments that improve body image, 4.1 times as much utility as treatments that reduce long‐term complications (e.g., 5/20 to 3/20) and 8.9 times utility for treatments that improve sexual wellbeing. As the highest level for acute side effects (8/20 having one) is zero, we were unable to calculate the relative importance.

The study was also able to calculate how much of one attribute a patient is willing to sacrifice in order to get more of another, for example, a patient may be willing to accept a higher risk of acute or long‐term complications to improve their life expectancy. In our study, we planned to assess trade‐offs in terms of two different attributes of treatment—the risk of acute side effects and long‐term complications.

We had originally planned to assess trade‐offs relating to overall survival, assigning continuous variables to survival (i.e. 5% increments)—but feedback from the patient focus groups indicated that the use of moderate and strong was preferable.

As trade‐offs can only be calculated using statistically significant coefficients, in our study, it was only possible to use data for long‐term complications for the analysis (*p* = <0.001). We defined chronic complications as consequences occurring months to years post treatment. In our study, patients would accept 100% risk of developing at least one chronic long‐term condition for a strong increase in life expectancy (and a 50% risk for a moderate increase). Body image was also important, and patients were willing accept a 16% risk of developing at least one chronic condition to avoid a significant change in body image (and a 13% risk for a slight change).

These trade‐offs for body image need to be interrupted in a broader sense, as it is unclear whether this relates to physical appearances (scars, skin changes or stoma related changes) or the ability to achieve bladder preservation to maintain quality of life. Other studies have shown that quality of life (defined as daily functioning, standard of health and comfort) was the most important preference within older patient cohort with a range of solid tumours (colorectal, breast, anal, gastrointestinal), followed by overall survival and disease‐free survival: Transient short‐term side effects were again deemed the least important consideration when undergoing cancer treatment.^1^ Knowing that patients prioritise survival above other outcomes, but risk of long‐term complications and changes to body image are also important, it is vital that patients are fully informed of all potential treatment related risk. In the United Kingdom, RC (+/− neoadjuvant chemotherapy) with urinary diversion and radical chemoradiotherapy (+/− neoadjuvant chemotherapy) are both considered standard of care management options for patients with organ confined MIBC. A subset of patients can also be considered for partial cystectomy (PC). RC is commonly recommended for MIBC but is associated with a high risk of post‐operative complications and relatively high mortality rates (compared to other treatments for MIBC) in the months following it; overall mortality rates range 0.8%–8%.^20^ Recent surveillance, epidemiology, and end results (SEER) database evaluations have highlighted the advantages of PC in highly selective patients in terms of minimised side effects with similar oncological outcomes compared to RC when combined with adequate lymph node dissection.^21^, ^22^, ^23^ Trimodality therapy (TMT) is also well tolerated and a viable alternative for select patients who wish to retain their bladder.^24^ Patient and tumour factors are important in the decision‐making process—but accurate presentation of outcome data relating to survival (both from cancer and management complications), acute toxicity (requiring hospital admission) and impact on body image are important for patients to make fully informed decisions. Direct comparison of these attributes for each intervention (neoadjuvant chemotherapy [NAC]/RC + continent diversion/RC + incontinent diversion/PC/radical radiotherapy/radical chemoradiotherapy) would provide patients with the information most important to them when discussion treatment preferences.

There are limitations to analysing DCEs. For example, the subjective interpretation of attributes by participants can be influenced by the language used. It therefore becomes important to be unambiguous in the definitions for each attribute. It is also important to interpret the findings considering the varied units of measurement for different attributes (quantitative vs. qualitative). For two of our attributes, namely, acute side effects and sexual wellbeing, the preference magnitudes did not follow a monotonic trend, meaning the intermediate level was preferred compared to the higher level of change and when calculating how much of a risk of developing a long‐term complication to improve sexual wellbeing, participants were willing to accept a higher risk for a worse outcome. This could be due to the loss of statistical power rather than a true reflection of participants' choices or perhaps it might be that the baseline sexual function was already poor and therefore sexual wellbeing was less of a concern for our participants. Due to our sample size, we were unable to stratify our findings based on patient age, cTNM or treatment status. We sought to determine attribute preferences in a fit population undergoing radical treatment; however, bladder cancer incidence rises with age and is often diagnosed in patients with limited functional reserve, frailty and comorbidities.^25^ It would also be important to undertake a DCE in this population to determine which factors drive their treatment choices, and a multi‐site DCE with a larger recruitment number would help understand the trade‐offs in more detail. Hence, there were limitations regarding our sample, in that it was recruited from a single site and was a relatively small sample size for a DCE. We were only able to approach 36% of the eligible patients over our recruitment period. Reasons for not approaching patients included recent ‘bad news’, perceived ‘information overload’ and logistic reasons during outpatient visits.

## 5 CONCLUSION

When deciding on the type of treatment, people with MIBC consider that survival is the most important factor, followed by the risk of chronic complications and changes to body image. Changes to sexual wellbeing and the risk of acute side effects did not reach statistical significance in our study. Regarding trade‐offs, our study highlighted that patients were willing to accept a higher risk of long‐term complications to improve life expectancy and body image. Understanding patient preferences is important for shared decision‐making, which has an impact on quality of care for people living with MIBC.

## AUTHOR CONTRIBUTIONS

*Design*: Liam Mannion, Simon Hughes and Vinod Mullassery. *Data collection*: Liam Mannion, Simon Hughes, Vinod Mullassery, Margaret Northover, Deborah Enting, Kate Smith, Kathryn Chatterton, Suzanne Amery, Rajesh Nair, Muhammad Shamin Khan and Ramesh Thurairaja. *Data analysis*: Liam Mannion and Verity Watson. *Writing*: All. *Proofreading*: All.

## CONFLICT OF INTEREST STATEMENT

Simon Hughes is an American Society of Clinical Oncology: Education Council member and British Uro‐oncology Group: Trustee and Committee member. All other authors declare no conflict of interest.

## Appendix A Factors relevant to treatment decisions for muscle invasive bladder cancer (MIBC) patients—literature review results

**Table:**

Author	Year of publication	Findings (summary)	Factors relevant to treatment decisions
Merten et al.	2019	The multimodal treatment consisted of a maximal TURBT followed by RT; concomitant platinum‐based chemotherapy combined with RHT in patients with high‐grade bladder cancer improves local control, bladder‐preservation rate and OS. It offers a promising alternative to surgical therapies like radical cystectomy.	• Survival • Organ preservation
Gergelis et al.	2019	Definitive RT +/− CT is a safe, effective and well‐tolerated treatment strategy for elderly patients with MIBC.	• Elderly patients >70 years tolerate TMT • Complications
James	2018	This lack of data supporting a survival advantage for surgery does not stop its proponents presenting it as the gold standard. It is, however, more likely that survival in bladder cancer is driven by the presence or absence of distant spread at the time of local therapy and will not be affected by the means adopted for local control. Furthermore, all patients undergoing surgery will need reconstructive bladder surgery. Thus, there are many patients for whom radical surgery is simply not suitable, and hence, bladder‐preserving techniques are appropriate. Radiotherapy should thus always be given, wherever possible, with a simultaneous radio‐sensitiser, the most robust data with UK fractionation being with 5FU/MMC or the BCON schedule.	• Survival • Distant metastases (recurrence)
Perez‐Montero et al.	2017	According to our data, TMT offers survival and local control rates comparable to modern RC series with the important advantage of bladder function preservation with low rates of salvage cystectomy. This modality should be offered as an alternative to RC in selected patients. The most appropriate cases for TMT are those with T2 R0 N0 disease.	• Survival • Complications
Stokes et al.	2017	OS did not significantly differ between SCC and UCC patients undergoing organ preservation for MIBC, whilst other prognostic factors were relevant in both groups. Limited prevalence and rare utilisation of organ preservation may have influenced these results. Further work is needed to define the optimal therapeutic strategy for MIBC‐SCC in Western countries.	• Histology variants • Survival
González et al.	2017	Organ preservation treatment of muscle invasive bladder cancer by TURBT and definitive RT or radiochemotherapy is feasible and effective, but it is necessary to make a correct selection of the patients.	• Patient selection • Organ preservation
Chen	2014	Not every patient with muscle invasive bladder cancer needs to undergo radical surgery and lose their bladder and adjacent organs. Similar to multiple other cancers, certain patients with bladder cancer can be offered organ‐preserving treatment, which is effective and safe.	• More info is needed to inform patients on the use of TMTs (complications) • Survival outcomes • Organ preservation

*Note*: Merten R, Ott O, Haderlein M, Bertz S, Hartmann A, Wullich B, et al. Long‐Term Experience of Chemoradiotherapy Combined with Deep Regional Hyperthermia for Organ Preservation in High‐Risk Bladder Cancer (Ta, Tis, T1, T2). Vol. 24, The Oncologist. Hoboken, USA: John Wiley & Sons, Inc; 2019. p. e1341–50. Gergelis KR, Kreofsky CR, Choo CS, Lester SC, Viehman J, Pisansky TM, et al. Organ Preservation with Definitive Radiotherapy for Elderly Patients with Muscle‐Invasive Bladder Carcinoma. Vol. 105, International Journal of Radiation Oncology, Biology, Physics. Elsevier Inc; 2019. p. E251–2. James N. SP‐0020: Organ preservation in bladder cancer—an evidence‐based alternative to radical surgery. Vol. 127, Radiotherapy and Oncology. Elsevier B. V; 2018. p. S7–S7. Perez‐Montero H, Bonel AC, Fasano M, Pedraza S, Guardado S, Mendoza AMC, et al. Long‐Term Outcomes of Organ Preservation for Bladder Cancer in a Large Cohort. Vol. 99, International Journal of Radiation Oncology, Biology, Physics. Elsevier Inc; 2017. p. E259–E259. Stokes WA, Kessler ER, Wilson S, Lam ET, Flaig TW, Kavanagh BD, et al. Organ Preservation for Muscle‐Invasive Squamous Cell Carcinoma of the Urinary Bladder in the United States. Vol. 99, International Journal of Radiation Oncology, Biology, Physics. Elsevier Inc; 2017. p. E266–E266. González E, Garduño S, Villanego I, Salas C, Gutierrez L, Macías MJ, et al. Organ preservation in muscle‐invasive bladder cancer. Vol. 16, European Urology Supplements. 2017. p. e2799. Chen RC. Organ Preservation—Will Data Translate into Reality for Bladder Cancer Patients?. Vol. 27, Clinical Oncology. England: Elsevier Ltd; p. 133–5.

Abbreviations: 5FU, fluorouracil; BCON, bladder carbogen and nicotinamide radiotherapy; CT, chemotherapy; MMC, mitomycin C; OS, overall survival; RC, radical cystectomy; RHT, regional deep hyperthermia; RT, radiotherapy; SCC, squamous cell carcinoma; TMT, trimodality therapy; TURBT, transurethral resection of a bladder tumour; UCC, urothelial cell carcinoma.
